# Genome-wide identification, molecular characterization, and gene expression analyses of honeysuckle NHX antiporters suggest their involvement in salt stress adaptation

**DOI:** 10.7717/peerj.13214

**Published:** 2022-04-19

**Authors:** Luyao Huang, Zhuangzhuang Li, Chunyong Sun, Shijie Yin, Bin Wang, Tongyao Duan, Yang Liu, Jia Li, Gaobin Pu

**Affiliations:** 1 Shandong University of Traditional Chinese Medicine, Jinan, China; 2 Ocean University of China, Qingdao, China

**Keywords:** Honeysuckle, Genome-wide, Na^+^/H^+^ antiporter (NHX), Salt stress

## Abstract

**Background:**

Ion homeostasis is an essential process for the survival of plants under salt stress. Na^+^/H^+^ antiporters (NHXs) are secondary ion transporters that regulate Na^+^ compartmentalization or efflux reduce Na^+^ toxicity and play a critical role during plant development and stress responses.

**Methods and Results:**

To gain insight into the functional divergence of NHX genes in honeysuckle, a total of seven LjNHX genes were identified on the whole genome level and were renamed according to their chromosomal positions. All LjNHXs possessed the Na^+^/H^+^ exchanger domain and the amiloride-binding site was presented in all NHX proteins except *LjNHX4*. The phylogenetic analysis divided the seven NHX genes into Vac-clade (*LjNHX1*/*2*/*3*/*4*/*5*/*7*) and PM-clade (*LjNHX6*) based on their subcellular localization and validated by the distribution of conserved protein motifs and exon/intron organization analysis. The protein-protein interaction network showed that *LjNHX4*/*5*/*6*/*7* shared the same putatively interactive proteins, including *SOS2*, *SOS3*, *HKT1*, and *AVP1*. Cis-acting elements and gene ontology (GO) analysis suggested that most LjNHXs involve in the response to salt stress through ion transmembrane transport. The expression profile analysis revealed that the expression levels of *LjNHX3*/*7* were remarkably affected by salinity. These results suggested that LjNHXs play significant roles in honeysuckle development and response to salt stresses.

**Conclusions:**

The theoretical foundation was established in the present study for the further functional characterization of the NHX gene family in honeysuckle.

## Introduction

Soil salinization is one of the major environmental stress that reduces plant growth and productivity throughout the world (Huang et al., 2019a; Nedjimi, 2016; Rengasamy, 2017). When plants are exposed to a high-salt environment, Na^+^ will enter the cells through the plasma-membrane non-selective ion channels (NSCCs) and high-affinity K^+^ transporter-1 (*HKT1*) protein, resulting in ionic toxicity and osmotic stress (Chen et al., 2015; Deinlein et al., 2014; Dong et al., 2021). The regulation of osmosis and ion homeostasis of cells under salt stress, especially the compartmentalizing and excluding ability of Na^+^, mainly depends on the activity of ion transporters and channels. In particular, the monovalent cation/proton antiporters (CPA) superfamily is one of the most important families in plant responses to salt stress, including Na^+^/H^+^ antiporters (NHXs), K^+^-efflux antiporters (KEAs), and cation/H^+^ exchangers (CHXs) and has been well characterized (Jia et al., 2018). The NHX family which belongs to the CPA1 family is expected to have 10–12 transmembrane domains while KEAs and CHXs are predicted to have 8–14 transmembrane domains, all of which contain the Na^+^/H^+^ exchanger domain (PF00999) (Wang et al., 2020b). These proteins function in regulating cation and pH homeostasis by exchanging Na^+^, K^+^, Li^+^ for H^+^ in plants, animals, fungi, and bacteria, and are mainly localized within the vacuole, plasma, and organelle membranes (Bassil et al., 2019; Sharma, Taneja & Upadhyay, 2020).

It was reported that the NHX family is conserved across various evolutionary lineages, which indicates that they play a vital role in the development of organisms (Sharma, Taneja & Upadhyay, 2020). NHXs participate in various biological processes such as salt tolerance, pH and ion balance regulation, cell expansion, stomatal function, cellular vesicle trafficking, protein processing, and flower development (Tian et al., 2017; Zhou et al., 2016). The eight NHX genes from *Arabidopsis* were divided into vacuole (Vac) -clade (*AtNHX1*, *AtNHX2*, *AtNHX3*, and *AtNHX4*), endosomal (Endo) -clade (*AtNHX5*, *AtNHX6*), and plasma membrane (PM) -clade (*AtNHX7*/ *SOS1*, *AtNHX8*), respectively, according to their subcellular location (Cui et al., 2020). Phylogenetic analysis showed that BvNHXs (*Beta vulgaris*) and GbNHXs (*Gossypium barbadense*) also clustered into three subclades, among which Vac-clade NHXs are the most abundant from all the studied species (Akram et al., 2020; Wu, Wang & Li, 2019). The function of NHX transporters can be preliminarily defined by their subcellular localization (Akram et al., 2020). For example, Vac-binding *AtNHX1* and *AtNHX2* function in controlling vacuolar K^+^ and pH homeostasis to regulate cell expansion, stomatal conductance, and floral organ development (Bassil et al., 2011b). Endomembrane-bounded *AtNHX5* and *AtNHX6* are involved in maintaining organelle pH and ion homeostasis with implications in endosomal sorting and cellular stress responses (Bassil et al., 2011a). *AtNHX8* is located on the PM and has been proved to be a Li^+^/H^+^ antiporter (Meng & Wu, 2018; Wang et al., 2013).

Many studies have provided convincing evidence for the involvement of the NHX gene in salt tolerance. In *Arabidopsis*, *AtNHX1*-*6* transport either Na^+^ or K^+^ into the vacuole or endosome in exchange for H^+^ efflux to the cytoplasm to maintain the cellular ion homeostasis (Barragan et al., 2012; Bassil & Blumwald, 2014; Bassil et al., 2011a). Different studies have shown that plants have stronger salt stress tolerance when *AtNHX1*, *AtNHX2*, *AtNHX3*, *AtNHX5*, or *AtNHX6* were overexpressed, or *AtNHX4* was deficient (Jia et al., 2018; Li et al., 2009; Liu et al., 2008; Wu et al., 2016); the *nhx5 nhx6* double-knockout mutant aborted the transport through the tonoplast, increasing the sensitivity to salt stress (Bassil et al., 2011a). PM-binding *AtNHX7*/ *SOS1* endowed plants with salt tolerance through the Ca^2+^-dependent SOS (Salt overly sensitivity) pathway, which is the core mechanism of plant salt tolerance (Dong et al., 2021; Ji et al., 2013). The SOS signaling pathway consists of SOS3, SOS2, and SOS1. Salt stress triggers cytosolic Ca^2+^ elevation that activates SOS3. SOS3 recruit SOS2 to the PM and activate its kinase activity. The SOS3-SOS2 complex targets the PM-localized Na^+^/H^+^ exchanger SOS1 to regulate ion transport processes at the PM (Deinlein et al., 2014; Ma et al., 2019). *AtNHX7* also plays a role in the long-term transport of Na^+^ (Hamam et al., 2016; Ji et al., 2013). In Arabidopsis, Qiu et al. found that plasma membrane Na^+^/H^+^ exchange activity was reduced by 80% in the *Atsos1* mutants compared to the control (Jia et al., 2018; Qiu et al., 2002). Overexpression of *AtSOS1* increased salt tolerance in transgenic *Arabidopsis* by reducing Na^+^ content in xylem and shoot (Yang et al., 2009).

Honeysuckle (*Lonicera japonica* Thunb.) belongs to the Caprifoliaceae family, is native to eastern Asia, and is now grown in many countries, such as Australia and the United States (Cai et al., 2021b; Pu et al., 2020). Its dried flower buds, leaves, and stems have been prescribed in traditional Chinese medicine (TCM) to treat fever, influenza, sores, and swelling for more than 1,500 years. Moreover, the benefits of honeysuckle have been demonstrated in the treatment of many emerging diseases such as influenza A viruses (H1N1, H5N1, H7N9), SARS coronavirus, hand-foot-and-mouth disease, and the early-stage novel coronavirus infection (Liu et al., 2005; Pu et al., 2020; Shang et al., 2011). It is believed to be an ecologically invasive species in several countries including New Zealand, Australia, Argentina, Mexico, and much of the USA because of its high environmental adaptability (Uddin et al., 2021). Previous studies have found that honeysuckle is highly resistant to salt stress, but the physiological and molecular mechanisms remain unclear (Cai et al., 2021b; Huang et al., 2019b). The analysis of LjNHXs in honeysuckle would enable a more comprehensive understanding of molecular mechanisms underlying Na^+^ homeostasis and plant salt stress resistance.

In this study, seven NHX genes were identified from the genome of *Lonicera japonica* and classified into Vac-clade and PM-clade based on their subcellular localization. The physicochemical properties, phylogenetic relationship, architecture of conserved motifs, gene structures, cis-acting elements of LjNHX genes, the distribution of LjNHX genes on chromosomes, protein tertiary structure, and putative protein-protein interaction (PPI) were comprehensively analyzed. Furthermore, we investigated the expression pattern of LjNHX family genes in the *Lonicera japonica* cultivar “Huajin 6” in different tissues and gradient salt stress. Our results could serve as a theoretical reference for an in-depth analysis of the response mechanisms of LjNHX family genes to salt stress and their mediation of plant resistance/tolerance to high salinity.

## Materials and Methods

### Identification of NHX genes in honeysuckle genome

The published NHX gene sequences of Arabidopsis and *Oryza sativa* were downloaded from the Arabidopsis Information Resource database (http://www.arabidopsis.org) and the Rice Genome Annotation Project (http://rice.plantbiology.msu.edu//), respectively. Further, the NHX genes were used as queries to search against *Lonicera japonica* genome databases to identify NHX genes from honeysuckle (Dong et al., 2021). Then, the Na^+^/H^+^ exchanger domain (PF00999) of honeysuckle NHX was confirmed using NCBI Conserved Domain Database (CDD, https://www.ncbi.nlm.nih.gov/Structure/cdd/wrpsb.cgi) and InterProscan (http://www.ebi.ac.uk/Tools/pfa/iprscan/) databases (Liu et al., 2019). Finally, Expert Protein Analysis System (ExPASy, http://web.expasy.org/compute_pi/) was used to predicate the isoelectric point (pI) and molecular weight (MW) (Bassil et al., 2019 ; Bjellqvist et al., 1993). TMHMM Server v2.0 (https://services.healthtech.dtu.dk/service.php?TMHMM-2.0) was used to predicate the transmembrane helices (TMHs) (Moller, Croning & Apweiler, 2001) and Plant-mPLoc (http://www.csbio.sjtu.edu.cn/bioinf/plant-multi/) was used to predicate subcellular localization of NHX protein sequences (Chou & Shen, 2010). The phosphorylation sites in the LjNHX proteins were predicted using NetPhos 3.1 Server. (https://services.healthtech.dtu.dk/service.php?NetPhos-3.1) (Blom, Gammeltoft & Brunak, 1999; Joshi et al., 2021).

### Phylogenetic analysis

The protein sequences of all the identified NHXs from seventeen angiosperms were aligned using the Clustal-Omega (https://www.ebi.ac.uk/services) (Wu, Wang & Li, 2019), including five monocots: *O. sativa* (Os, seven sequences), *Sorghum bicolor* (Sb, six sequences), *Triticum aestivum* (Ta, three sequences), *Panicum virgatum* (Pv, one sequence), *Hordeum vulgare* (Hv, four sequences), and twelve eudicots: *Lonicera japonica* (Lj, seven sequences), *Arabidopsis thaliana* (At, eight sequences), *Populus trichocarpa* (Pt, eight sequences), *Vitis vinifera* (Vv, eight sequences), *Solanum lycopersicum* (Sl, seven sequences), *Cucurbita maxima* (Cm, five sequences), *Gossypium hirsutum* (Gh, fifteen sequences), *Salicornia europaea* (Se, one sequence), *Beta vulgaris* (Bv, five sequences), *Eutrema halophilum* (Eh, five sequences), *Spinacia oleracea* (So, five sequences), *Solanum tuberosum* (St, five sequences). The software MEGA 6 was used to construct a phylogenetic tree of 100 NHXs using the Maximum likelihood (ML) method. The bootstrap value was 1,000 replicates (Kumar, Stecher & Tamura, 2016). All the sequences of NHX proteins were presented in Table S1. The EMBOSS needle (https://www.ebi.ac.uk/Tools/psa/) was used to calculate the pairwise identity and similarity of proteins (Tian et al., 2017).

### Conserved motifs, gene structures, and cis-acting elements analysis

The conserved motifs of the NHX protein sequence from honeysuckle were predicted using the Multiple Expectation Maximization for Motif Elicitation program (MEME version 5.0.5, http://meme-suite.org/tools/meme) (Bailey et al., 2009). The exon/intron structure of NHX proteins was graphically displayed by the Gene Structure Display Serve (GSDS, http://gsds.cbi.pku.edu.cn/) based on the genomic sequences (Hu et al., 2015). For cis-acting regulatory elements, the 2,000 nucleotide sequences upstream of the transcription initiation site were predicted and analyzed using PlantCARE database (http://bioinformatics.psb.ugent.be/webtools/plantcare/html/) (Lescot et al., 2002; Wu, Wang & Li, 2019).

### Chromosome location and Ka/Ks ratio analysis

The chromosome distribution of the NHX genes was mined from the annotation information on the honeysuckle genome database and gene distribution visualized with TBtools (Chen et al., 2020; Wu, Wang & Li, 2019). The MCScanX program (https://github.com/tanghaibao/mcscan) was used to identify the gene duplication events (Wang et al., 2012). The synonymous (Ks) and non-synonymous (Ka) substitution of each duplicated gene pair were calculated using the PAL2NAL program (http://www.bork.embl.de/pal2nal/) (Goldman & Yang, 1994).

### Protein tertiary structure prediction

The tertiary structure of NHX proteins was predicted using the I-TASSER program (https://zhanglab.ccmb.med.umich.edu/I-TASSER/) (Yang et al., 2015). LOMETS was a multiple threading approach that could be used to identify the best structural templates from the Protein Data Bank (PDB) database (Berman et al., 2003; Wu & Zhang, 2007).

### Protein-protein interaction network analysis

The protein-protein interaction (PPI) network of LjNHX proteins was predicted using a model plant *Arabidopsis* on STRING protein interaction database (STRING, http://string-db.org) (Szklarczyk et al., 2019).

### Plant material, treatment, and qRT-PCR analysis

The salt-tolerant honeysuckle cultivar ‘Huajin 6’ was used as the material for pot cultivation in the greenhouse of Shandong University of Traditional Chinese Medicine Medicinal Botanical Garden (Huang et al., 2021; Huang et al., 2019b). Five tissues including mature leaf, young leaf, flower, stem, and root of 2-year-old honeysuckle were collected for tissue-specific expression analysis in June 2021. To verify the genes regulated by salt stress, the annual seedling of honeysuckle was transplanted to plastic containers filled with quartz sand/vermiculite (1/3) in April 2021. After three months, the seedlings were treated with the mixed solution containing 1/2 Hoagland’s nutrient solution and NaCl (0, 100, 200, or 300 mM) solution. Roots were collected from the seedlings at 0, 3, 6, 12, 24, 48, and 72 h after treatments for RT-qPCR analysis.

Total RNA was extracted from the samples using a FastPure Plant Total RNA Isolation Kit (Vazyme, Beijing, China). RNA was reverse transcribed to cDNA using PrimeScript RT reagent Kit with gDNA Eraser (TaKaRa, Shiga, Japan). Primers for RT-qPCR were designed using Primer Premier 6 based on the CDS of genes with melting temperature of 58–62 °C. RT-qPCR analysis was performed using a CFX96 Real-Time System (BIO-RAD, Hercules, CA, USA) with TB Green Premix Ex Taq II (TaKaRa, Shiga, Japan). The relative expression level of LjNHXs in different tissues and gradient salt stress was calculated by the 2^–ΔCt^ and 2^–ΔΔCt^ methods, respectively (Wang et al., 2020a; Zhu et al., 2016). The data were subjected to analysis of variance with Tukey’s multiple range tests means at a significant level of *P* < 0.05 using the SPSS. Three replicates were performed for each sample. The primers used in this study are listed in Table S1.

## Results

### Identification of *LjNHX* genes

A total of eight NHX genes were finally obtained from the honeysuckle genome. These honeysuckle genes were named *LjNHX1*-*LjNHX7* according to their chromosomal positions. As shown in Table 1a, the physicochemical properties showed that the deduced amino acid lengths of LjNHXs were exhibited from 380 aa (*LjNHX4*) to 1,148 aa (*LjNHX6*), with an average length of 591 aa. The pI of the LjNHX proteins ranged from 5.88 to 8.15, with an average pI of 7.12. The predicted M.W of the LjNHX proteins ranged from 41.71 to 127.03 kDa, with an average M.W of 75.29 kDa. All the LjNHXs were typical transmembrane transporters, possessed the Na^+^/H^+^ exchanger domain. *LjNHX1*/*6*/*7* contained 12 transmembrane helices. *LjNHX2* contained 11 transmembrane helices. *LjNHX3*, *LjNHX5*, *LjNHX4* contained 10, 9, 5 transmembrane helices, respectively. The prediction of subcellular localization showed that all LjNHX proteins might be located in the vacuoles except *LjNHX6*, which could be found in the plasma membrane. LjNHXs phosphorylation sites vary in number (Table 1b), serine sites ranged from 25 (*LjNHX5*) to 87 (*LjNHX6*), threonine sites ranged from 15 (*LjNHX4*) to 39 (*LjNHX6*), while tyrosine sites ranged from zero (*LjNHX1*) to 9 (*LjNHX6*). LjNHXs were mostly phosphorylated with PKC, CdC2, and PKA, respectively, and very little with ATM.

**Table 1:** Characteristics of NHX genes identified from honeysuckle.

(a) Characteristics of NHX genes identified from honeysuckle.										
Gene Name	Gene ID	Chr	No. Amino acid	pI	Protein M.W (kDa)	Exon/Intron	Arabidopsis ortholog	TM domains	Subcellular localization	Na^+^/H^+^ exchanger domain
*LjNHX1*	Ljap00034208	2	512	7.72	56.05	13/13	At3g05030	12	Vac	84–407
*LjNHX2*	Ljap00014455	3	536	8.15	59.21	14/14	At3g05030	11	Vac	40–427
*LjNHX3*	Ljap00006626	4	536	6.78	58.94	14/13	At3g05030	10	Vac	52–439
*LjNHX4*	Ljap00011905	7	380	5.88	41.71	9/9	At3g05030	5	Vac	10–274
*LjNHX5*	Ljap00015571	8	503	7.71	55.48	14/15	At3g05030	9	Vac	51–398
*LjNHX6*	Ljap00035581	8	1148	5.93	127.03	23/22	At2g01980	12	PM	29–451
*LjNHX7*	Ljap00009930	9	522	7.65	58.32	13/12	At5g55470	12	Vac	53–439

(b) Phosphorylation sites of LjNHX																			
Protein name	Phosphorylation sites			PKC	CKII	RSK	PKA	UNSP	EGFR	INSR	PKG	CKI	DNAPK	CdC2	p38MAPK	CDK5	GSK3	ATM	SRC
	S	Th	Ty																
*LjNHX1*	35	16	0	13	2	0	7	15	0	0	0	5	1	6	2	1	1	1	0
*LjNHX2*	31	17	4	14	1	0	5	29	1	2	0	1	1	6	1	1	0	0	0
*LjNHX3*	32	21	1	16	1	0	4	23	0	0	0	0	2	5	1	1	0	0	0
*LjNHX4*	26	15	2	10	2	0	2	24	0	0	0	0	0	4	0	0	0	0	1
*LjNHX5*	25	19	2	15	2	0	4	15	1	0	0	0	1	5	1	2	0	0	0
*LjNHX6*	87	39	9	24	11	1	11	58	0	3	3	7	3	12	1	1	0	0	0
*LjNHX7*	30	13	4	11	0	1	5	23	0	0	0	0	1	5	1	0	0	0	0

**Note:**

PKC, protein kinase C; CKII, Casein kinase 2; RSK, Ribosomal S6 kinase; PKA, Protein kinase A; UNSP, Un secified phosphorylation; EGFR, Epidermal growth factor receptor; INSR, Insulin receptor precursor; PKG, Protein kinase G; CKI, Casein kinase 1; DNAPK, DNA dependent protein kinase; CDC2, Cell Division cycle protein 2; P38MAPK, P38 Mitogen activated protein kinase; CDK5, Cyclin dependant kinase 5; GSK3, Glycogen synthase kinase 3; ATM, Ataxia telangiectasia mutated; S, Serine; Th, Threonine; Ty, Tyrosine.

### Phylogenetic analysis

**Figure 1:**
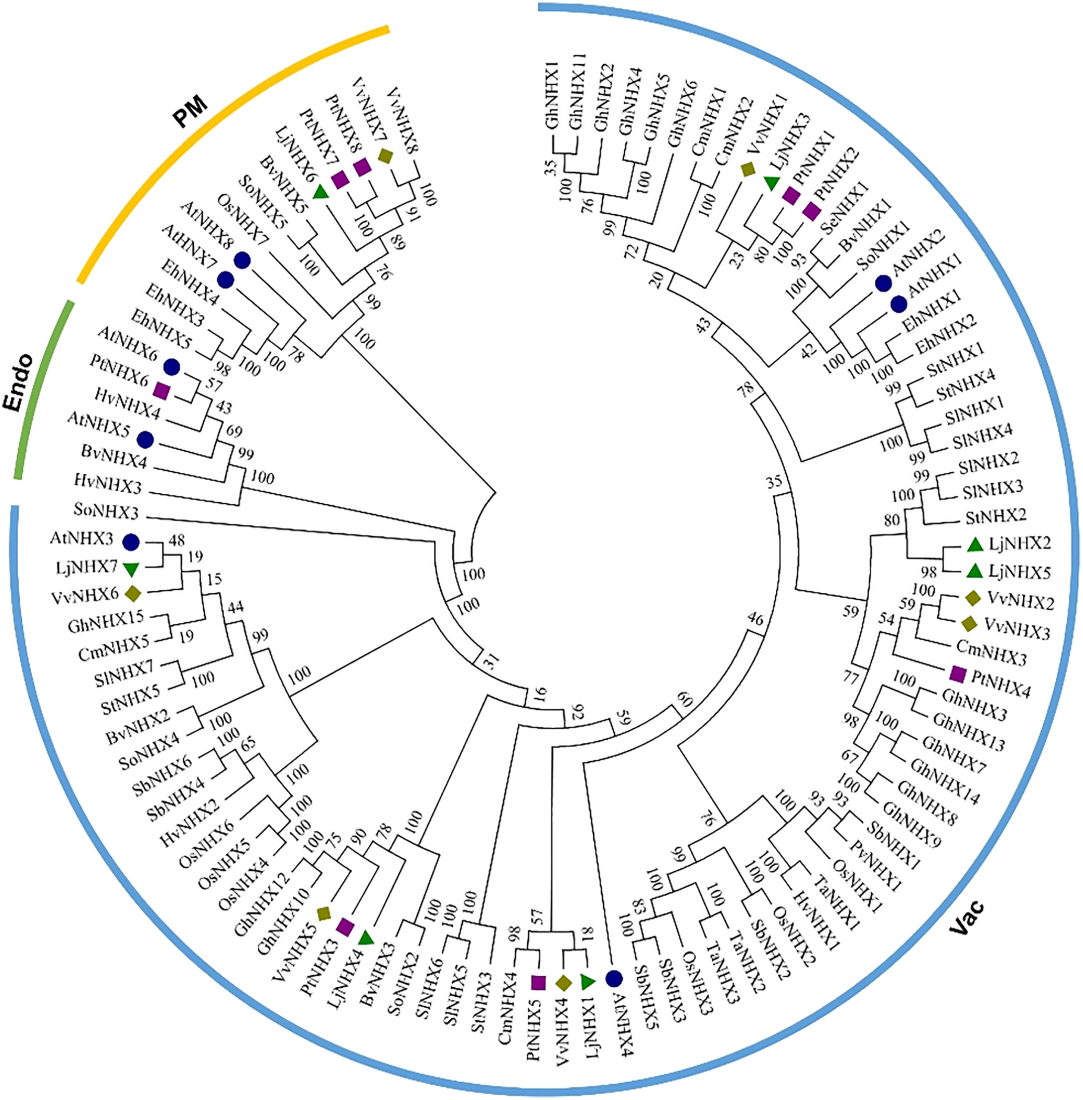
Phylogenetic tree of the NHX proteins. Phylogenetic tree of the NHX proteins from *O. sativa* (Os), *Sorghum bicolor* (Sb), *Triticum aestivum* (Ta), *Panicum virgatum* (Pv), *Hordeum vulgare* (Hv), and twelve eudicots *Lonicera japonica* (Lj), *Arabidopsis thaliana* (At), *Populus trichocarpa* (Pt), *Vitis vinifera* (Vv), *Solanum lycopersicum* (Sl), *Cucurbita maxima* (Cm), *Gossypium hirsutum* (Gh), *Salicornia europaea* (Se), *Beta vulgaris* (Bv), *Eutrema halophilum* (Eh), *Spinacia oleracea* (So), *Solanum tuberosum* (St). The different colored arcs indicate different clades. Proteins from honeysuckle, Arabidopsis, grape, and poplar are denoted by green triangles, blue circles, yellow diamonds, and purple squares, respectively. Details of sequences from seventeen species are listed in Table S1.

The phylogenetic tree (Fig. 1) divided all the 100 NHX proteins into three clades based on their predicted subcellular localization, Vac (vacuolar membrane), Endo (endomembrane), and PM (plasma membrane). There were 85 proteins in the Vac-clade, indicating that most types of NHX proteins from different species are vacuolar membrane-bound, while the PM-clade contained 13 proteins and Endo-clade contained 6 proteins. Among the honeysuckle, the Vac-clade had the largest number of members, with seven LjNHX (*LjNHX1*/*2*/*3*/*4*/*5*/*7*) proteins, PM-clade had only one protein (*LjNHX6*). Putatively, no NHX protein belongs to Endo-clade in honeysuckle.

The conservation of the sequence of NHX genes was also confirmed by the identities and similarities of amino acid sequences (Table 2). The results showed that the amino acid sequence identity of different LjNHXs ranged from 8.7% to 78.9%, while the amino acid sequences similarity ranged from 13.8% to 83.6%. The sequences of *LjNHX2*/ *LjNHX5* have higher identities (78.9%), which were members of a close evolutionary relationship. Overall, the genes that belong to the same clades exhibit higher identities.

**Table 2:** Pairwise sequence similarity and identity among LjNHX and AtNHX proteins.

Identity (%)																
	Protein names	*LjNHX1*	*LjNHX2*	*LjNHX3*	*LjNHX4*	*LjNHX5*	*LjNHX6*	*LjNHX7*	*AtNHX1*	*AtNHX2*	*AtNHX3*	*AtNHX4*	*AtNHX5*	*AtNHX6*	*AtNHX7*	*AtNHX8*
Similarity (%)	*LjNHX1*		68.2	66.2	36.4	63.3	10.5	49.6	63.0	64.2	53.4	59.3	26.8	25.0	11.4	14.7
	*LjNHX2*	76.3		76.6	44.9	78.9	10.0	53.2	73.4	73.9	56.7	66.2	30.0	27.3	12.5	15.4
	*LjNHX3*	76.3	84.7		42.7	72.6	10.6	54.9	74.8	76.1	56.7	66.2	30.1	26.7	12.4	16.9
	*LjNHX4*	46.1	53.2	51.5		41.4	8.7	36.8	42.2	41.4	37.7	36.0	20.8	19.7	8.6	12.8
	*LjNHX5*	72.4	83.6	80.5	48.3		9.8	50.7	69.9	69.7	52.3	62.2	26.2	25.4	11.9	16.4
	*LjNHX6*	17.8	17.9	18.1	13.8	18.4		10.8	10.6	11.1	10.5	10.5	10.7	12.3	60.1	42.4
	*LjNHX7*	62.8	66.4	69.6	49.5	61.8	18.6		53.9	53.8	68.8	51.9	27.8	26.0	11.6	15.7
	*AtNHX1*	74.5	84.0	86.4	51.1	79.8	18.3	68.7		87.4	56.5	65.2	29.3	28.8	12.2	16.5
	*AtNHX2*	75.8	83.3	86.1	51.1	79.3	18.7	67.4	92.9		56.3	65.3	29.7	28.9	12.0	16.9
	*AtNHX3*	66.7	70.7	72.6	48.3	66.5	18.3	78.6	71.6	71.2		54.4	28.9	27.3	11.3	16.5
	*AtNHX4*	68.5	75.5	76.5	44.2	69.6	17.5	64.7	74.4	75.9	68.0		30.1	26.7	11.3	14.9
	*AtNHX5*	42.1	45.5	46.8	33.0	41.2	18.8	45.4	45.0	45.4	46.5	46.4		76.4	11.7	14.6
	*AtNHX6*	41.0	44.0	43.7	32.6	42.4	21.2	42.5	46.6	45.1	44.3	44.2	84.9		11.9	17.9
	*AtNHX7*	20.5	21.2	20.9	14.7	19.8	73.5	20.8	21.2	21.1	20.8	20.1	19.5	19.7		48.3
	*AtNHX8*	27.9	26.5	28.9	22.5	28.6	51.1	29.0	28.9	30.3	29.0	25.6	26.1	31.2	56.1	

### Chromosomal location, Ka/Ks ratio calculation of *LjNHX* genes

**Figure 2:**
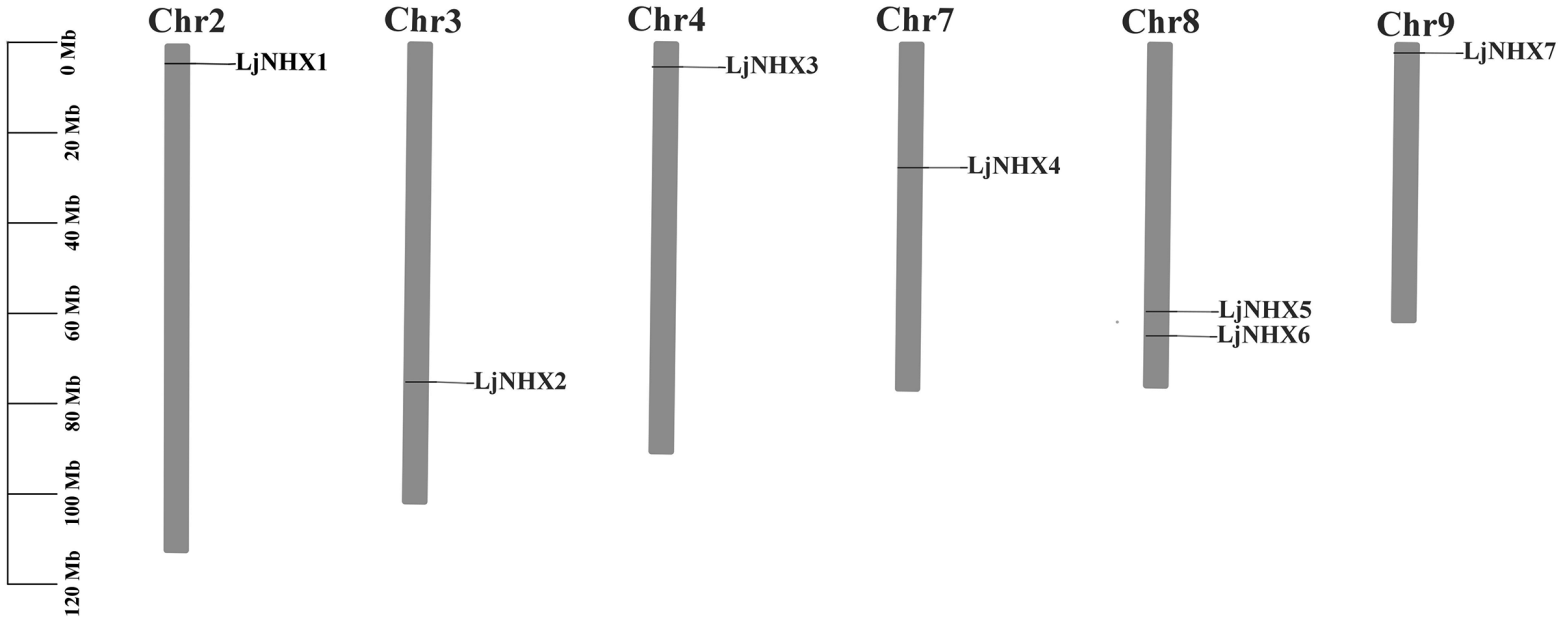
Physical mapping of honeysuckle NHX genes. The chromosome number is displayed at the top of each chromosome.

As shown in Fig. 2, seven LjNHX genes were mapped onto six of the total nine honeysuckle chromosomes, indicating a diverse distribution. Chromosome 8 contained two members of LjNHXs, while chromosomes 2, 3, 4, 7 and 9 each contained only one LjNHXs gene. In this study, however, only one duplicated gene pair (*LjNHX2*/*LjNHX5*) was identified, and the gene pair was located on different chromosomes (Table S1). The genes had undergone strong purifying selection pressure because the Ka/Ks ratio between *LjNHX2* and *LjNHX5* is 0.0979 (Wu, Wang & Li, 2019).

### Gene structure and conserved motifs analysis

**Figure 3:**
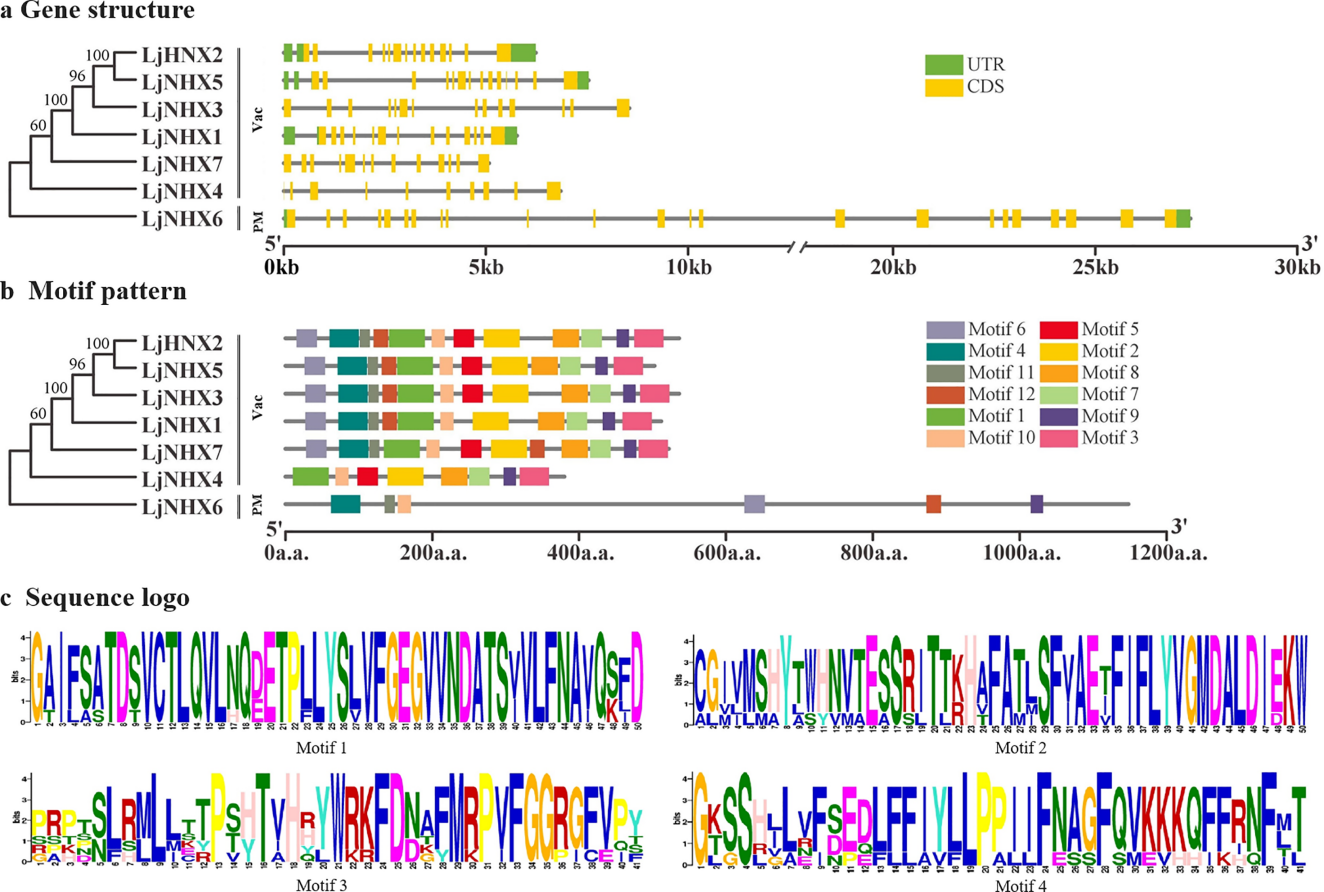
Phylogenetic relationships, gene structure and motif pattern of LjNHXs. (A) Exon-intron structure. (B) Motif composition. (C) Sequence logo of motif 1, 2, 3, 4. The sequence information and sequence logo for each motif is provided in Table S1 and Fig. S1, respectively.

The intron-exon structure of the identified LjNHX genes showed that the coding sequence (CDS) of all the LjNHXs was discontinuous by the presence of introns (Fig. 3A). *LjNHX1*/*2*/*3*/*5*/*7* in Vac-clade contained 13–14 exons and 12–15 introns, but the *LjNHX4* contained 9 exons and 9 introns. *LjNHX6* in PM-clade had 23 exons and 33 introns. Overall, although gene length differed significantly among the NHX gene family members, the exon length, exons/intron number are moderately conserved among the various subclades, indicating the similar biological functions of members with close evolutionary relationships.

A total of 12 putative motifs were identified in NHX proteins, which represent the characteristic region of proteins (Fig. 3B). Here, motif 9/10 existed in all the members of NHX proteins, whilst motif 1/2/3/7/8 existed in all members of Vac-clade. Amiloride-binding site, a typical feature of NHX protein, was presented in motif 4. Motif 4 was presented in all NHX proteins except *LjNHX4*. However, there were differences in the composition of amiloride-binding sites. All proteins in Vac-clade were FFIYLLPPI, while LLAVFLPALL was in PM-clade.

### Cis-acting elements located in promoters of *LjNHXs*

In the NHX promoters (Table 3), five hormone-related (*e.g.*, ABRE, TCA-element, CGTCA-motif, TGACG-motif, TGA-motif), seven stress-related (*e.g.*, ARE, LTR, MBS, TC-rich repeats, W box, WUN-motif, STRE), and eight development-related (*e.g.*, G-box, GT1-motif) elements were identified (Wu, Wang & Li, 2019). All of the members contained hormone-related elements, except *LjNHX2*. *LjNHX6* contained four types of hormone-related elements, including ABA (abscisic acid), SA (salicylic acid), MeJA (methyl jasmonate) and IAA (auxin)-responsive element. *LjNHX7* contained 14 stress-response elements, which was the largest number of hormone-related elements contained within one gene. Among stress-related cis-acting elements, ARE was found in all LjNHX promoters, while WUN-motif and STRE were found in most LjNHX promoters. *LjNHX6* contained six types of stress-related elements, while *LjNHX2*/*3* contained five types of stress-related elements. Additionally, light-responsive elements were found in all LjNHX promoters. These results implied that LjNHX genes could be involved in hormone signal responsiveness and stress adaptation.

**Table 3:** Kinds and amounts of hormone-, stress-, and development-related cis-acting element.

Functional class	Elements	Function	Genes						
			*LjNHX1*	*LjNHX2*	*LjNHX3*	*LjNHX4*	*LjNHX5*	*LjNHX6*	*LjNHX7*
Hormone	ABRE	ABA-responsive element	0	0	4	0	2	2	9
	TCA-element	Salicylic acid-responsive element	1	0	1	2	0	1	0
	CGTCA-motif	MeJA-responsive element	0	0	0	0	0	1	2
	TGACG-motif	MeJA-responsive element	0	0	0	0	0	1	2
	TGA-element	Auxin-responsive element	0	0	1	1	0	0	0
Stress	ARE	Anaerobic induction	1	2	3	2	1	1	1
	LTR	Low-temperature responsiveness	2	0	1	0	0	3	0
	MBS	MYB binding site involved in drought-inducibility	1	1	1	0	7	0	0
	TC-rich repeats	Defense and stress responsive element	1	0	0	0	0	1	1
	W box	WRKY Transcription factor binding site	0	3	0	1	0	2	0
	WUN-motif	Wound-responsive element	0	1	1	1	1	2	1
	STRE	Stress response element	0	3	1	4	2	1	2
Others	MYB	Transcription factor	5	4	8	1	9	2	4
	G-box	Light-responsive element	0	0	4	0	1	3	10
	GT1-motif	Light-responsive element	1	2	2	0	1	3	3
	TCT-motif	Light-responsive element	1	1	1	1	2	0	0
	Box 4	Light responsiveness	3	2	1	4	2	1	2
	CAT-box	Meristem expression	1	0	1	1	0	0	0
	GCN4_motif	Endosperm expression	0	1	0	0	0	0	0
	O2-site	Zein metabolism regulation	0	0	0	0	0	1	0

### Protein tertiary structure

**Figure 4:**
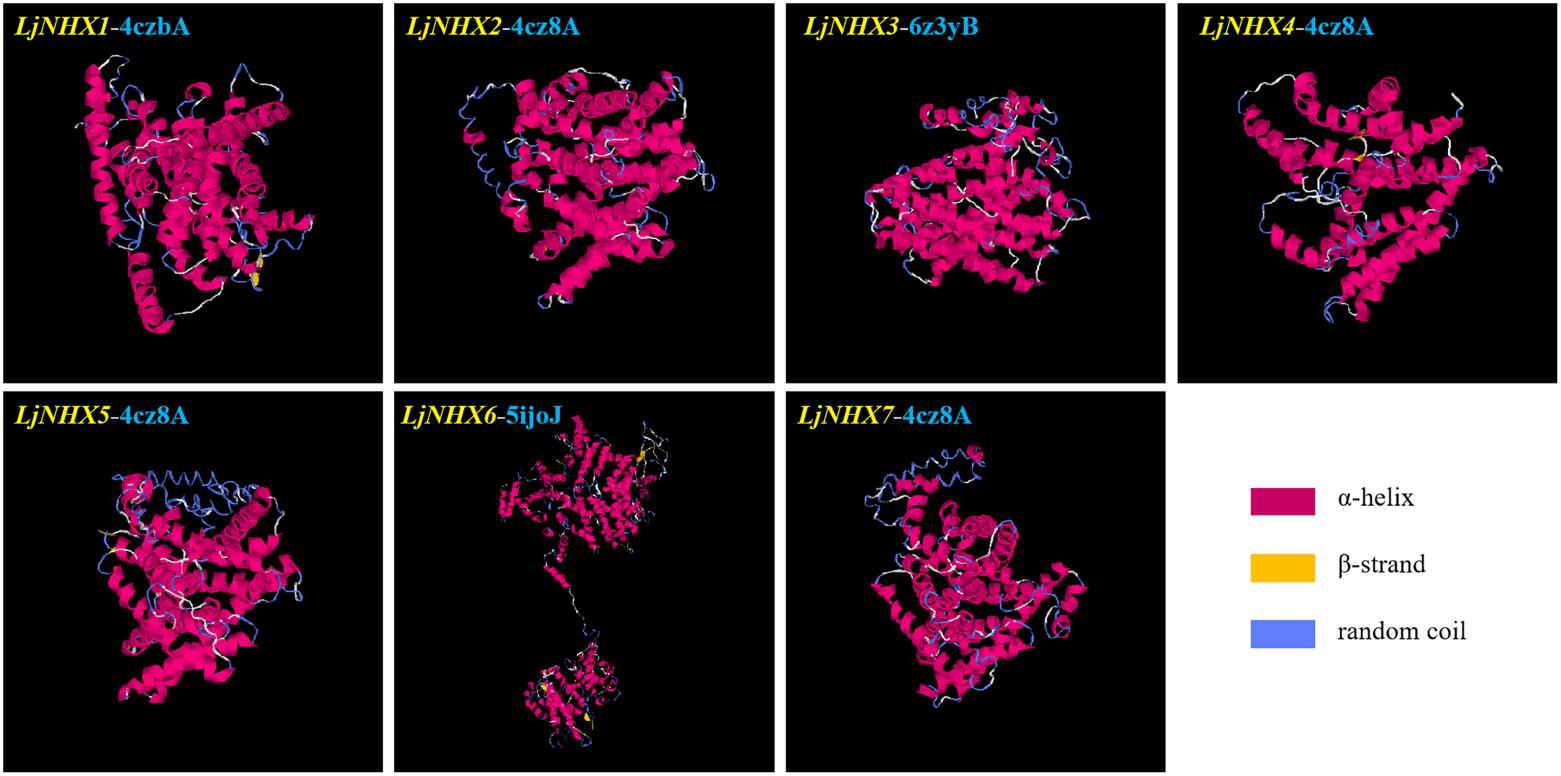
The tertiary structure of seven LjNHX proteins. Details of the secondary structure of LjNHXs are shown in Fig. S2.

Tertiary structures of LjNHX protein (Fig. 4) were construed based on the ideal structural templates and crystal structures from Protein Data Bank (PDB) (Tian et al., 2017; Wu, Wang & Li, 2019). All the predicted NHX models in this study had a C-score varied from −1.79 (*LjNHX1*) to −0.17 (*LjNHX7*), suggesting the structures of LjNHXs were constructed with high credibility (Table 4). Among them, *LjNHX 2*/*4*/*5*/*7* shared the same PDB hit 4cz8A, indicating that their tertiary structures were similar. Therefore, we speculated that *LjNHX 2*/*4*/*5*/*7* has similar biological functions.

**Table 4:** Structural dependent modeling parameters for the NHX proteins.

Protein	C-score	TM-score	RMSD (Å)	Best identified structural analogs in PDB				
				PDB hit	TM-score	RMSD	IDEN	Cov
*LjNHX1*	−1.79	0.50 ± 0.15	11.6 ± 4.5 Å	4czbA	0.722	0.68	0.177	0.727
*LjNHX2*	−0.25	0.68 ± 0.12	8.0 ± 4.4 Å	4cz8A	0.719	1.19	0.207	0.731
*LjNHX3*	−1.38	0.54 ± 0.15	10.7 ± 4.6 Å	6z3yB	0.708	1.1	0.312	0.718
*LjNHX4*	−0.92	0.60 ± 0.14	8.8 ± 4.6 Å	4cz8A	0.697	1.31	0.21	0.716
*LjNHX5*	−1.7	0.51 ± 0.15	11.4 ± 4.5 Å	4cz8A	0.717	1.69	0.201	0.742
*LjNHX6*	−0.90	0.60 ± 0.14	11.4 ± 4.5 Å	5ijoJ	0.922	1.41	0.076	0.931
*LjNHX7*	−0.17	0.69 ± 0.12	7.8 ± 4.4 Å	4cz8A	0.727	1.26	0.194	0.741

**Note:**

C-score [−5, 2] is the confidence of each model, a higher value indicates a model with higher confidence and vice-versa. TM-score and RMSD are determined based on the C-score value and the protein length following the correlation observed between these qualities. TM-score indicates a measure of global similarity between query structure and known structure in PDB. RMSD represents the RMSD between residues that are structurally aligned by TM-align. IDEN is the percentage sequence identity in the structurally aligned region. Cov is the coverage of the alignment by TM-align and is equal to the number of structurally aligned residues divided by length of the query protein.

### Protein-protein interaction network analysis

**Figure 5:**
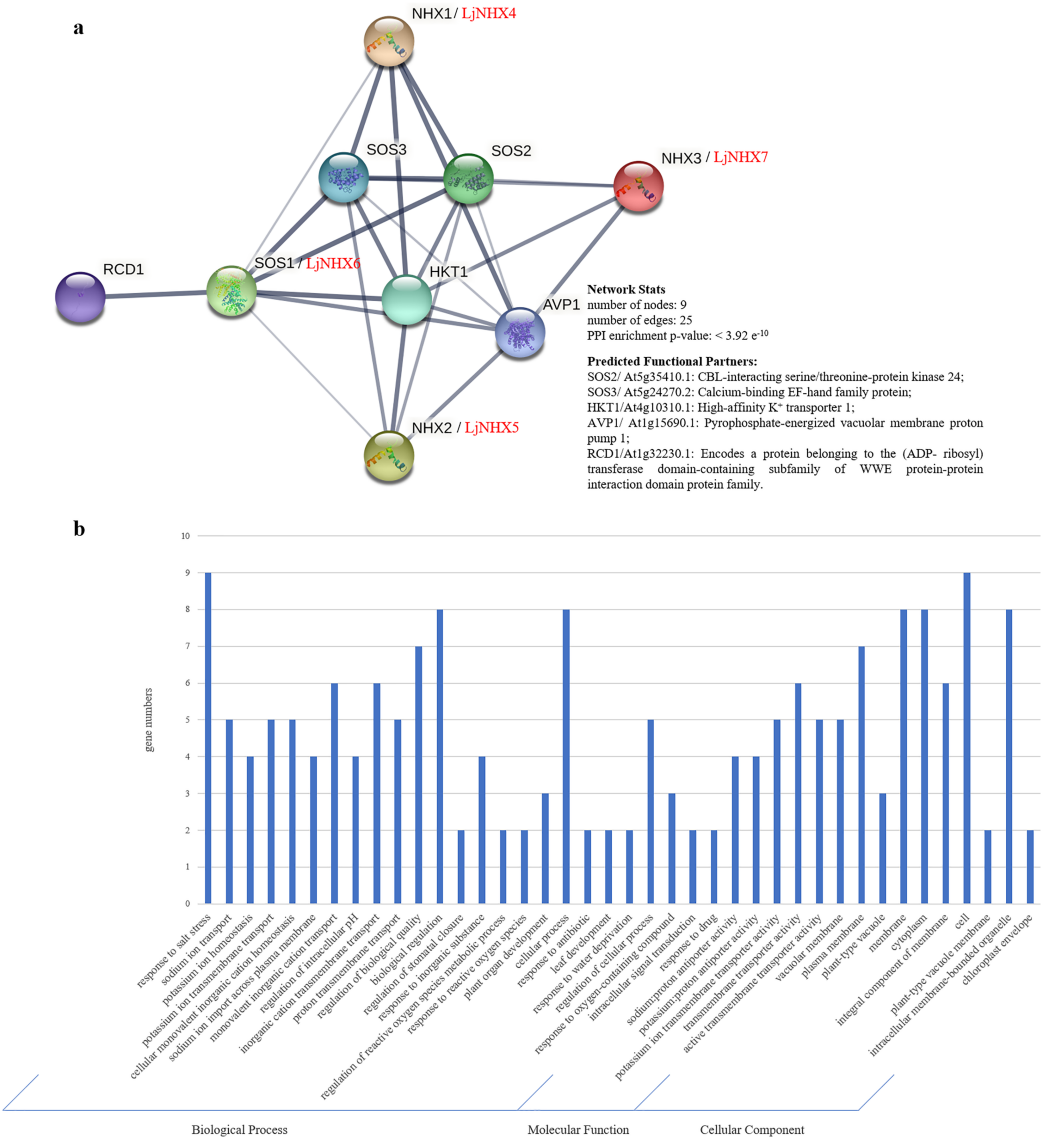
Protein-protein interaction (PPI) prediction of LjNHXs. (A) PPI network. Line thickness indicates the strength of data support. (B) Gene Ontology (GO) analysis of the genes from the PPI network.

Protein-protein interaction (PPI) network was constructed using STRING database to further explore the potential functions of LjNHX during their interactions with other cellular proteins based on either known experimental or predicted interactions. On the STRING, *Lonicera japonica* PPI network is not available until now, therefore, we used the homolog gene between *Arabidopsis thaliana* and *Lonicera japonica* to predict the LjNHX PPI network (Bassil et al., 2011a). As shown in Fig. 5A, *LjNHX4*/*5*/*6*/*7* shared the same putatively interactive proteins, including *SOS2*, *SOS3*, *HKT1*, and *AVP1*. *LjNHX6* interacts with five proteins, such as *HKT1*, conferring salinity tolerance and *RCD1*, which supports chloroplasts against high ROS (Reactive oxygen species). *SOS2* and *SOS3* were involved in the regulatory pathway of salt stress by controlling intracellular Na^+^ and Ca^2+^ homeostasis and directly interacted with *LjNHX4*/*5*/*6*/*7* proteins. All predicted LjNHX proteins worked together to respond to salt stress.

Gene Ontology (GO) analysis (Fig. 5B) was used to describe the proteins from the interaction network, including molecular function (MF), biological processes (BP), and cellular components (CC). It has been shown that the proteins were mainly localized to the plasma membrane, vacuolar membrane, and cytoplasm. Regarding MF, most of the proteins possess transmembrane transporter activity, including sodium: proton transporter activity and potassium: proton transporter activity. In the BP process, most of the proteins mainly participate in the response to salt stress and play a role in ion transmembrane transport and ion homeostasis.

### Expression patterns of *LjNHXs*

**Figure 6:**
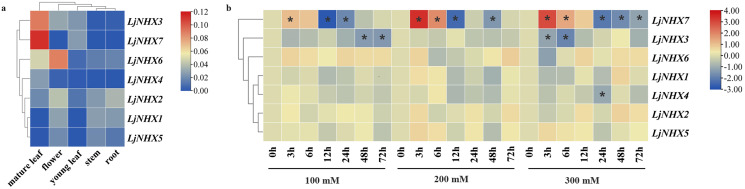
Expression patterns of LjNHX genes. (A) Expression patterns of LjNHX in five tissues. All values were expressed relative to the expression levels of reference genes using formula 2^−ΔCt^ (Wang et al., 2020a). (B) Expression patterns of the LjNHX at 0, 3, 6, 12, 24, 48, and 72 h after treated with NaCl (100, 200, and 300 mM NaCl). All values were expressed relative to the expression levels of reference genes using formula 2^−ΔΔCt^. *Ljactin* was used as a marker gene. Different colors indicate different levels of gene expression based on the log2 value of the fold change by RT-qPCR. The asterisk indicates significant (*P* < 0.05) up/down-regulated expression (>2-fold).

For tissue-specific expression analysis (Fig. 6A), the examined genes were expressed in all selected tissue under normal conditions, although their expression levels differed among tissues. *LjNHX3/7* had a high level of expression in the mature leaf, while *LjNHX6* exhibited a high level of expression in the mature leaf and flower. *LjNHX2* had a high level of expression in root and flower. Our results showed that LjNHX genes may play important roles in the growth and development of honeysuckle, and for which functional variations are probable.

The expression pattern of NHX genes under salinity stress was illustrated in Fig. 6B, the expression of *LjNHX7* under all NaCl treatments rapidly increased at the first time and then decreased. The highest expression of *LjNHX7* appeared at 3 h, while the lowest expression appeared at 12 h (100 and 200 mM NaCl) and 24 h (300 mM NaCl), respectively. *LjNHX3* decreased its expression level at 3 h and maintained a lower expression level over time. Notably, when exposed to low salt stresses (100 mM NaCl), the expression of *LjNHX3* reached the lowest level at 72 h, but its expression reached the lowest level at 6 h under high salt concentration (300 mM NaCl).

## Discussion

Transmembrane ion transport is a critical process in the cellular response to salt stress. NHXs are secondary ion transporters to exchange H^+^ and transfer the Na^+^ or K^+^ across the membrane, playing an essential regulatory role in maintaining intracellular pH and ion balance (Farooq et al., 2015; Rubio, Gassmann & Schroeder, 1995). Honeysuckle plays an irreplaceable role in the development of TCM (Traditional Chinses Medicine) (Li et al., 2019b; Tang et al., 2021) and has the potential to grow in saline-alkaline soil (Cai et al., 2021a). The whole-genome sequence of the honeysuckle was completed, which made it possible to analyze the NHX gene families of honeysuckle using bioinformatics.

Identification and characterization of the NHX family have been widely reported in some plant species, but the number of members is varied. A total of seven NHX genes were identified in honeysuckle based on the Na^+^/H^+^ exchanger domains. There are eight NHX genes in *Arabidopsis thaliana* (Maser et al., 2001), six in *Vitis vinifera* (Ma et al., 2015), eight in *Populus trichocarpa* (Tian et al., 2017), and 25 in *Gossypium barbadense* (Akram et al., 2020). The difference in the number of NHX genes among different species is due to gene duplication and loss events during evolution (Wu, Wang & Li, 2019). Duplication events provide opportunities for the generation of new genes and their functional divergence in the process of gene family expansion and evolution. As a consequence, the paralogous genes were generated (Tian et al., 2017). The paralogous pair (*LjNHX2*/*LjNHX5*) in honeysuckle was generated by segmental duplication, as the genes are present on different chromosomes (Akram et al., 2020). The Ka/Ks ratio gives an insight into the selection pressure on amino acid substitutions. A Ka/Ks ratio > 1 suggests positive selection, Ka/Ks ratio = 1 shows neutral selection, while the ratio of Ka/Ks < 1 suggests purifying selection (Bassil et al., 2011a; Nekrutenko, Makova & Li, 2002). The result from *LjNHX2*/*LjNHX5* pair revealed that LjNHX gene family was strongly purified and selected during long-term evolution and is functionally conserved (Akram et al., 2020).

Most members of the NHX family contain 10–12 transmembrane structures, about 550 amino acid residues, and a putative amiloride-binding domain (FFI/LY/FLLPPI) in the third transmembrane region (Joshi et al., 2021; Ma et al., 2015). But, not all of the members have these characteristics. *LjNHX5* and *AtNHX5* have nine transmembrane structures, while *LjNHX4* only has five transmembrane structures; *LjNHX6* has 1,148 aa residues and *AtNHX7*/*SOS1* has 1,146 aa residues. All except *LjNHX4* possess an amiloride-binding site in the N-terminal. It has been shown that the presence of amiloride even in a micro concentration in the Na^+^/H^+^ antiporters inhibits the transport of Na^+^ (Bassil et al., 2011a; Carmen & Larry, 1999; Counillon, Franchi & Pouyssegur, 1993). The site was found in most Na^+^/H^+^ antiporters of plants, such as *Vitis vinifera* and *Arabidopsis*, implying that the site on the transmembrane region of LjNHXs is sensitive to the Na^+^ of the substrate (Ma et al., 2015; Qiu et al., 2004). However, Ma et al. (2015) showed that the position of this domain was not conserved, and many NHXs genes don’t contain the amiloride-binding domain, including the VIT_15s0024g 00280 in grapevine (Ma et al., 2015).

The phylogenetic analysis serves as an excellent method to determine evolutionary relationships and functional associations among genes. Phylogenetic analysis of NHX genes in honeysuckle, together with five monocots and twelve eudicots, classified in total 100 NHXs into three different clades according to their vacuolar, endosomal, and plasma membrane localization. Similar clustering has also been found in poplar (*Populus trichocarpa*) (Tian et al., 2017) and Sugar Beet (*Beta vulgaris*) (Wu, Wang & Li, 2019). In general, subcellular localization is an effective reference for defining the function of the NXH transporter. Consistent with the results from the prediction of TMHMM Server, the phylogenetic analysis of honeysuckle divided the LjNHXs into Vac-clade (*LjNHX1*/*2*/*3*/*4*/*5*/*7*) and PM-clade (*LjCIPK6*). NHX members located on the PM function in the exclusion and compartmentalization of excess Na^+^, while the endomembrane-bounded NHXs are essential for cellular cargo trafficking, growth development, and the regulation of protein processing (Akram et al., 2020; Bassil et al., 2011a). However, no gene was identified in the Endo-clade, indicating an uneven distribution of NHXs among subclades and species. Additionally, consistent with the current information of plant evolution, the phylogenetic tree of LjNHXs was more closely related to eudicots, especially grape (*Vitis vinifera*) and poplar (*Populus trichocarpa*), compared to monocots. The results of similar exon/intron and motifs patterns within the same subclade further support the accuracy of the phylogenetic tree (Cui et al., 2020). However, the differences in motif patterns among members of the same subclade cannot be ignored. And the structural and motif differences between different subclades reveal the functional diversity of the NHX gene family in honeysuckle (Dong et al., 2021).

As the binding sites of transcription factors, cis-acting regulatory elements play an important role to determine genes’ expression patterns (Wu, Wang & Li, 2019). Hormones, such as ABA, SA, MeJA, and IAA, are essential for every stage of plant development and response to stresses (Gallego-Giraldo et al., 2008; Li et al., 2019a; Mishra et al., 2014; Zhang & Li, 2019). ABA is a common mediator of plant responses to abiotic stress like high salt, low water and high temperature (Nakashima & Yamaguchi-Shinozaki, 2013). ACGT-containing ABA-responsive elements (ABREs, PyACGTGG/TC) were identified upstream for the most LjNHXs, and similar to that detected for many ABA and abiotic stress-inducible genes (Guiltinan, Marcotte & Quatrano, 1990; Hobo et al., 1999; Skriver et al., 1991), indicating that the LjNHXs might be involved in the ABA signal pathway, which mainly controls stomatal closure and physiological responses to salinity, drought, and cold stress (Mishra et al., 2014). TCA-element associated with biotic and abiotic stress was found in *LjNHX1*/*3*/*4*/*6*. Seven stress-responsive elements were identified namely W box, ARE, MBS, TC-rich repeats, LTR, WUN-motif, STRE, suggesting that LjNHXs responded to many kinds of stresses. For example, STRE elements are activated by heat shock, osmotic stress, low pH, nutrient starvation, etc. ARE is induced by anaerobiosis, and TC-rich repeats play a role in response to defense and stress (Tong et al., 2021). Among them, W box (TTGACC) was identified in *LjNHX 2*/*4*/*6*. W box is recognized by the family of WRKY transcription factors, which is involved in certain developmental processes and stress response (Wu, Wang & Li, 2019), such as salt stress response in *Populus* (Jiang et al., 2020) and *Arabidopsis* (Xu et al., 2018). Consistent with this, the expression of multiple genes of LjNHXs was significantly up/down-regulated under NaCl treatment. In short, NHX genes contains a variety of cis-elements related to stress and hormone response, indicating that the NHX family plays a key role in the process of honeysuckle coping with stress.

It was reported that NHXs are involved in salt tolerance responses of different species, such as *Gossypium barbadense* (Akram et al., 2020) and *Glycine max* (Joshi et al., 2021). Our study revealed that in honeysuckle, the NHX genes express differentially at different time intervals under different salt stress intensities. As a key pathway for plants to maintain intracellular ion homeostasis, the SOS pathway is composed of *SOS3*, *SOS2* and *SOS1* (Ji et al., 2013; Liu et al., 2020). Pairwise sequence alignment results showed that the sequences of *LjNHX6* and *AtNHX7* (At2g01980) have higher identities. PM-bounded *LjNHX6* was predicted to interact with *SOS3* and *SOS2* in the PPI network and *LjNHX6* possesses several hormone- and stress-related cis-acting elements, which indicated the key role of *LjNHX6* proteins in the exclusion of Na^+^ ions from the cell. Besides, as shown in Fig. 6B, expression profile analysis revealed that *LjNHX6* showed a higher expression in roots under almost all treatments. The up-regulation of SOS signaling pathway genes in response to salt stress has been confirmed in a variety of plants including poplar (*Populus trichocarpa*) (Tang et al., 2010) and spinach (*Spinacia oleracea*) (Zhao, William & Sandhu, 2020). These results collectively illustrate the conservation of SOS pathway genes in honeysuckle, and at the same time, prove that the SOS pathway is a common but necessary pathway for regulating plant salt stress resistance (Zhao, William & Sandhu, 2020), and the detailed mechanism is still to be explored.

The putative interactions between *LjNHX4*/*5*/*6*/*7* and *HKT1*, *AVP1* might play essential roles in the salt tolerance of honeysuckle. The GO analysis showed that these genes are involved in sodium ion transport across the membrane and salinity response activities. *LjNHX7* showed a unique pattern of first increase and then decrease under salt stress. *HKT1* participates in the recirculation of Na^+^ from above ground to the root system, thereby preventing Na^+^ from accumulating to a toxic level in shoots (Deinlein et al., 2014). In *Puccinellia tenuiflora*, the *HKT1;5* is strongly expressed in a high salt environment to increase the salinity tolerance by unloading excess Na^+^ from the xylem (Bassil et al., 2011a; Zhang et al., 2017). *AVP1* participates in the regulation of extracellular pH and auxin transport (Dong et al., 2021). Shen et al. (2014) found that the salt tolerance of transgenic cotton co-overexpressed *AVP1* and *AtNHX1* was improved. The expression profile analyses revealed that *LjNHX4* was downregulated under salt stress. In PPI network, *LjNHX4* protein was also hypothesized to interact with *RCD1*, and *RCD1* might play a significant role in response to high salt or oxidative stress. In Arabidopsis, *SOS1* interacts with *RCD1* to increase the tolerance against oxidative stress caused by ROS (Surekha Katiyar-Agarwal et al., 2006). In addition, a high number of stress-related cis-acting elements was observed in promoters of *LjNHX4*/*5*/*6*/*7*, so we hypothesized that there is an interaction between *LjNHX4*/*5*/*6*/*7* and *HKT1*, *AVP1, RCD1* to participate in the salt stress response of honeysuckle.

## Conclusions

In the present study, a total of seven LjNHX genes were identified. The phylogenetic analysis divided LjNHX genes into two subclades based on their subcellular localization and the same clade had similar motif compositions and gene structures. Analysis of cis-acting elements showed that LjNHX members may all be involved in hormone signaling response and stress adaptation. PPI network analysis showed that *LjNHX4*/*5*/*6*/*7* shared the same putatively interactive proteins, including *SOS2*, *SOS3*, *HKT1*, and *AVP1*, and *LjNHX6* might be the primary Na^+^/H^+^ antiporter involved in the SOS pathway during the salt stress response. The GO analysis showed that these genes mainly participate in the response to salt stress and play a role in ion transmembrane transport and ion homeostasis. The salt-induced expression patterns confirmed that the expression levels of *LjNHX3/7* were remarkably affected by salinity. The systematic bioinformatics analysis indicates that the NHX family plays an important role in the response of honeysuckle to salt stress, and the results lay a foundation for gene transformation technology, to obtain highly salt-tolerant medicinal plants in the context of the global reduction of cultivated land.

## Supplemental Information

10.7717/peerj.13214/supp-1 Supplemental Information 1 Phylogeny sequences. Primer sequence. The non-synonymous/ synonymous (Ka/Ks) ratio. Motif sequences.

10.7717/peerj.13214/supp-2 Supplemental Information 2 Details of motifs in LjNHX proteins.

10.7717/peerj.13214/supp-3 Supplemental Information 3 Details of the secondary structure of LjNHX proteins.

10.7717/peerj.13214/supp-4 Supplemental Information 4 Raw data of qRT-PCR.

## Additional Information and Declarations

### Data Availability

The following information was supplied regarding data availability: The datasets are available in the article and the Supplemental Files. The raw sequencing data are available at NCBI GenBank: MZ796249–MZ796255.
