# Supplementary figures and images for: Genome-wide identification, molecular characterization, and gene expression analyses of honeysuckle NHX antiporters suggest their involvement in salt stress adaptation
Source: PeerJ. 2022 Apr 19;10:e13214. doi: 10.7717/peerj.13214 (PMC9029436; doi:10.7717/peerj.13214)

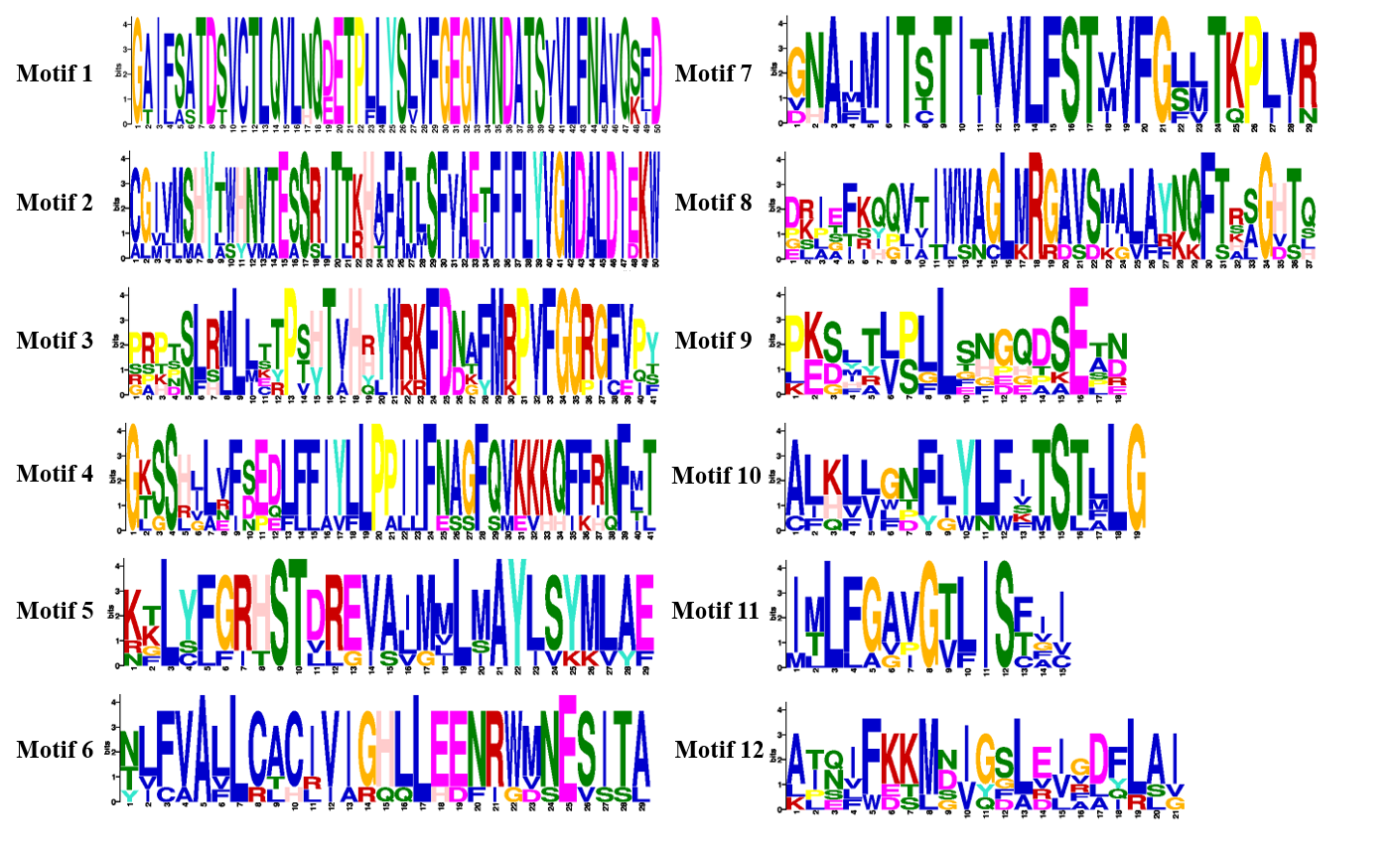

Supplement: Supplemental Information 2 [file peerj-10-13214-s002.png]

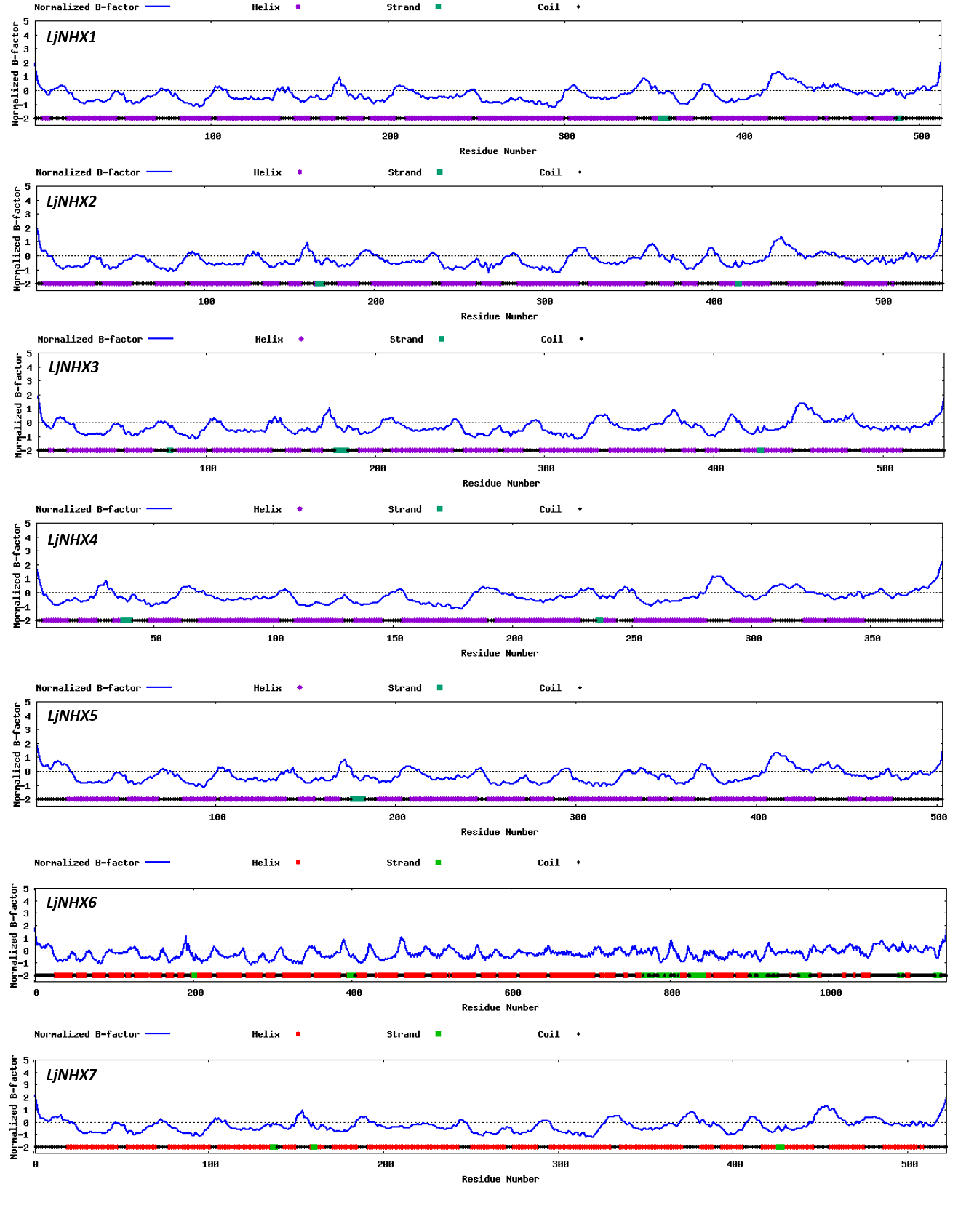

Supplement: Supplemental Information 3 [file peerj-10-13214-s003.png]
